# Major Histocompatibility Complex (MHC) and Fungal Antigen Presentation: Immunological Mechanisms and Their Role in Anti-Fungal Immunity

**DOI:** 10.3390/ijms27219533

**Published:** 2026-10-25

**Authors:** Fangyi Shu, Nopawit Khamto, Artid Amsri, Sirida Youngchim, Joshua D. Nosanchuk, Thinnaphop Chum-in, Qingping Deng, Kritsada Pruksaphon

**Affiliations:** 1 Department of Anatomy, School of Basic Medical Sciences, Youjiang Medical University for Nationalities, Baise 533000, China; shufangyi02@sina.com; 2 Department of Biochemistry, Faculty of Medical Science, Naresuan University, Phitsanulok 65000, Thailand; nopawitk@nu.ac.th; 3 Department of Medical Science, Mahidol University Amnatcharoen Campus, Amnatcharoen 37000, Thailand; artida076@gmail.com; 4 Department of Microbiology, Faculty of Medicine, Chiang Mai University, Chiang Mai 50200, Thailand; syoungchim@gmail.com; 5 Covenant HealthCare College of Medicine, Central Michigan University, Mount Pleasant, MI 48859, USA; josh.nosanchuk@einsteinmed.edu; 6 Department of Food Industry, School of Agricultural Technology and Food Industry, Walailak University, Nakhon Si Thammarat 80160, Thailand; thinnaphop.ch@mail.wu.ac.th; 7 School of Medical Imaging, Youjiang Medical University for Nationalities, Baise 533000, China; deng_040308@sina.com; 8 Department of Medical Technology, School of Allied Health Sciences, Walailak University, Nakhon Si Thammarat 80160, Thailand; 9 Research Center for Microbiome, Systems Biology and Medical Innovation (MiSys Center), Walailak University, Nakhon Si Thammarat 80160, Thailand

**Keywords:** major histocompatibility complex (MHC), MHC restriction, fungal immunogens, T-cell response, acquired immunity, antigen presentation, fungal vaccine

## Abstract

Cellular-mediated immunity against invasive fungal infections is fundamentally governed by Major Histocompatibility Complex (MHC) restriction, whereby T cells detect fungal invasion indirectly through peptide fragments displayed on host MHC molecules rather than by recognizing intact fungal pathogens. In contrast to B cells, which bind native microbial antigens, T lymphocytes monitor antigen-presenting cells for processed fungal peptides presented by MHC class I and class II molecules. This review examines the molecular mechanisms underlying fungal antigen processing and presentation, emphasizing the pivotal contributions of MHC class II-restricted CD4 T-helper (TH1/TH17) cells and cross-presented, MHC class I-restricted CD8 cytotoxic T lymphocytes to antifungal host defense. We also summarize key experimental approaches, including overlapping peptide libraries, ELISpot assays, and peptide–MHC multimers, for defining MHC function and identifying immunodominant fungal antigenic determinants. Evidence from representative pathogenic fungal antigens, including Asp f16, Crf1, Eng2, and the multivalent rCpa1 vaccine, highlights both the promise and complexity of achieving cross-protective cellular immunity. Importantly, amino acid sequence similarity alone is insufficient to predict T-cell cross-reactivity, indicating that computational prediction and experimental epitope mapping should be employed as complementary approaches for the accurate identification and validation of immunologically relevant epitopes. Finally, elucidating the molecular principles governing fungal peptide presentation establishes a rational foundation for the development of broadly protective epitope-based vaccines and next-generation immunotherapeutic strategies.

## 1. Introduction to Acquired Fungal Immunology

*“Unlike the B cell, though, the T cell isn’t looking for a culprit to come bursting out of the saloon, guns a-blazing. It is, like some omniscient Sherlock Holmes with a pipe and an umbrella, seeking the portent of a person.”*

The song of the cell: An exploration of medicine and the new human by Siddhartha Mukherjee (2022) [1].

The analogy presented in The Song of the Cell elegantly illustrates the fundamental distinction between B-cell and T-cell antigen recognition. B cells function like sentinels that directly recognize intact pathogens or native antigens through their surface immunoglobulin receptors, analogous to identifying a criminal who openly reveals themselves. In contrast, T cells never encounter intact microbes directly. Instead, they infer the presence of infection by examining short antigenic peptides displayed on major histocompatibility complex (MHC) molecules by host cells. Much like Sherlock Holmes reconstructing the identity of an unseen suspect from subtle clues, T cells deduce the existence of intracellular pathogens from peptide–MHC complexes rather than the pathogens themselves. This principle, known as MHC restriction, forms the foundation of acquired cellular immunity. In fungal infections, this concept is particularly relevant because T cells recognize fungal peptide–MHC complexes, rather than intact fungal cells or cell-wall components. Extracellular fungal antigens acquired by professional antigen-presenting cells are processed within endosomal–lysosomal compartments and presented on MHC class II molecules to activate CD4 T cells, whereas selected fungal antigens can also be cross-presented on MHC class I molecules to prime CD8 T cells. Consequently, antifungal T-cell immunity relies on interpreting peptide “signs” left by fungal pathogens, enabling precise immune surveillance and coordinated protective responses without direct recognition of the intact microorganism. A comprehensive understanding of fungal antigens, the peptide epitopes derived from these antigens, and the MHC alleles responsible for their presentation is a fundamental prerequisite for the rational development of targeted immunotherapies and epitope-based subunit vaccines. This review provides an in-depth examination of the experimental methodologies used to map these antigen MHC–TCR interactions, critically evaluates the evidence supporting MHC function in major fungal pathogens, and discusses the translational implications of these findings for the development of broadly protective antifungal vaccines and adoptive T-cell therapies [2,3].

Invasive fungal infections represent one of the most pressing medical challenges of the modern era, as highlighted by the WHO Fungal Priority Pathogens List (FPPL) published in 2022 [4]. Mycoses pose a particularly serious threat to immunocompromised individuals, including recipients of hematopoietic stem cell transplantation, solid organ transplant recipients, and patients undergoing intensive chemotherapy. Major opportunistic and endemic fungal pathogens—including *Aspergillus fumigatus*, *Candida albicans*, *Coccidioides* spp., *Blastomyces dermatitidis*, *Talaromyces marneffei*, and *Histoplasma capsulatum*—cause life-threatening diseases associated with substantial morbidity and high mortality rates [5]. Historically, host defense against these fungal pathogens was considered to rely predominantly on the innate immune system, particularly the antifungal activities of neutrophils and macrophages [6]. However, advances in immunological research have unequivocally demonstrated that the acquired immune system, orchestrated by diverse subsets of T lymphocytes, is indispensable for the effective clearance of fungal pathogens and the establishment of long-term protective immunity [7,8]. MHC, also known as the Human Leukocyte Antigen (HLA) system in humans, is a highly polymorphic genetic locus responsible for antigen presentation. MHC restriction is a fundamental principle of acquired immunity whereby T-cell receptors recognize antigenic peptides only when presented by self-MHC molecules. Antigen-presenting cells process exogenous proteins into peptide fragments that are loaded onto MHC class II molecules for recognition by CD4 T lymphocytes, thereby initiating antigen-specific immune responses. This mechanism is broadly applicable across infectious diseases and toxin-mediated immunity. For example, elapid α-neurotoxins and other three-finger toxins contain CD4 T-cell epitopes that are processed and presented via MHC class II, promoting T-cell activation, B-cell help, affinity maturation, and the generation of neutralizing antibodies [9]. Accordingly, the identification of immunodominant MHC-restricted epitopes provides valuable insights for the rational design of epitope-based vaccines and improved immunotherapeutic strategies [10].

The activation of T-cell mediated immunity against microbial pathogens is fundamentally governed by the immunological phenomenon known as MHC restriction. T-cell receptors (TCRs) are incapable of recognizing free peptides or intact, unprocessed proteins. Instead, they specifically recognize peptide fragments with defined amino acid sequences that are presented within the peptide-binding groove of MHC molecules expressed on the surface of professional antigen-presenting cells (pAPCs) [11]. This fundamental principle of acquired immunity determines the specificity, magnitude, and effectiveness of immune responses against fungal pathogens. CD4 T helper (TH) cells, which predominantly differentiate into the protective TH1 and TH17 subsets during antifungal immunity, recognize peptides presented by MHC class II molecules (MHC class II restriction) [12]. Conversely, CD8 cytotoxic T lymphocytes (CTLs), which contribute to antifungal defense through direct cytotoxic activity against infected host cells and the production of pro-inflammatory cytokines, recognize fungal peptide antigens presented by MHC class I molecules (MHC class I restriction). In the absence of CD4 T-cell help, exogenous fungal antigens can stimulate memory CD8 T cells through MHC class I–restricted mechanisms. Cytokines produced by CD8 T cells, including tumor necrosis factor-α, interferon-γ, and granulocyte–macrophage colony-stimulating factor, contribute to the establishment of durable vaccine-induced immunity. Furthermore, CD8 T cells can employ alternative protective mechanisms to maintain effective vaccine-mediated immunity even when one or more of these cytokine-dependent pathways are absent [13,14].

## 2. Recognition of Self and Altered Self by T-Cell Receptors: Central Tolerance and the Immunological Basis of MHC Restriction

The ability of T lymphocytes to discriminate between healthy host tissues and cells that have become infected or undergone malignant transformation is founded on the principle of MHC restriction, one of the central concepts of modern immunology. This principle was first demonstrated by Peter C. Doherty and Rolf M. Zinkernagel through their pioneering studies of CTLs responding to lymphocytic choriomeningitis viral infection [15,16]. Their work established that T cells recognize foreign antigens only when they are presented by self-MHC molecules. This discovery fundamentally transformed our understanding of acquired immunity and was recognized with the 1996 Nobel Prize in Physiology or Medicine [17]. Unlike antibodies, which bind directly to native antigens, TCRs recognize antigens exclusively in the form of processed peptide fragments presented by self-MHC molecules on the surface of APCs or infected target cells. The simultaneous recognition of both the antigenic peptide and the self-MHC molecule therefore constitutes the molecular foundation of cell-mediated immunity. The human immune system is built upon two complementary principles: the recognition of self and the recognition of altered self [18]. Evolution has not simply equipped the immune system to detect foreign molecules; rather, it has endowed it with the ability to recognize host cells that have been altered by infection, malignant transformation, or other pathological processes. This concept of altered self lies at the heart of immune surveillance against infectious diseases, cancer, and numerous disorders arising from intracellular abnormalities.

The capacity to distinguish self from altered self is established during T-cell development in the thymus. The mammalian immune system contains a diverse repertoire of TCRs generated through somatic V(D)J recombination, allowing T cells to recognize a wide range of foreign antigens. However, a defining feature of conventional αβ T cells is MHC restriction. During thymic development, T cells are selected to recognize antigenic peptides only in the context of self-MHC molecules; the same peptides presented by MHC molecules of a different haplotype from another individual are not recognized, as T cells fail to recognize antigens presented by non-self or allogeneic APCs [16]. This property ensures that TCR-mediated recognition is simultaneously antigen-specific and self-MHC-dependent, which underlies the biological basis of T cell antigen recognition. Because TCR generation is a largely stochastic process, some TCRs inevitably recognize self-peptides presented by self MHC molecules. Central tolerance helps prevent autoimmune responses by eliminating or modifying self-reactive T cells during their development in the thymus. In the meantime, MHC polymorphism contributes to differences in antigen presentation among individuals and increases the collective ability of human populations to respond to diverse microbial pathogens [19,20].

The thymus is the primary site of T cell development and central tolerance. It provides a specialized environment in which developing thymocytes are selected according to their ability to recognize self MHC molecules and their potential to react against self-antigens. T cell progenitors derived from hematopoietic stem cells in the bone marrow migrate to the thymus, entering near the corticomedullary junction. Their migration and development are regulated by chemokines, including the CCL25/CCR9 axis, which supports their movement through the thymic microenvironment [21]. Survival and differentiation also depend on signals from thymic stromal cells, including IL-7 and Delta-like ligand 4 (DLL4), which activates Notch signaling. In the thymic cortex, double negative (CD4^−^CD8^−^) thymocytes undergo TCR β chain gene rearrangement and β selection before developing into double positive (CD4^+^CD8^+^) thymocytes expressing functional αβ TCRs. These cells then undergo positive and negative selection, which determine the specificity and self-reactivity of the mature T cell repertoire.

Positive selection allows thymocytes capable of recognizing self MHC molecules to survive. Double positive thymocytes move through the cortex and interact with peptide MHC (pMHC) complexes presented by cortical thymic epithelial cells (cTECs). Cells whose TCRs fail to recognize these complexes sufficiently do not receive the signals required for survival and undergo apoptosis, a process known as death by neglect [22,23]. Thymocytes that recognize self pMHC complexes with low to moderate affinity receive survival signals and continue their development into single positive CD4+ or CD8+ T cells. This process establishes MHC restriction, ensuring that mature T cells can recognize antigens presented by the host’s own MHC molecules. Following positive selection, thymocytes increase their expression of CCR7, which guides their migration into the thymic medulla in response to CCL19 and CCL21. In the medulla, they encounter a wider range of self-antigens and undergo further selection to limit autoreactivity.

In contrast to positive selection, negative selection, or clonal deletion, removes thymocytes that recognize self-pMHC complexes too strongly. Although this process can occur in the cortex through interactions with dendritic cells, it takes place predominantly in the thymic medulla. Medullary thymic epithelial cells (mTECs) and dendritic cells present self-antigens to developing single positive thymocytes. Strong TCR recognition of these antigens can trigger intracellular signaling pathways that induce apoptosis, eliminating potentially autoreactive cells before they enter the peripheral circulation. Costimulatory molecules, including members of the B7 family, also influence thymocyte responses during this process. In addition, cytokines and intracellular signaling pathways help regulate thymocyte survival, differentiation, and apoptosis. These effects depend on the specific cytokine and cellular context and should not be considered equivalent mechanisms of clonal deletion. Together, positive and negative selection produce a mature T cell repertoire that recognizes foreign antigens presented by self MHC molecules while limiting the risk of autoimmune responses.

**Figure 1:**
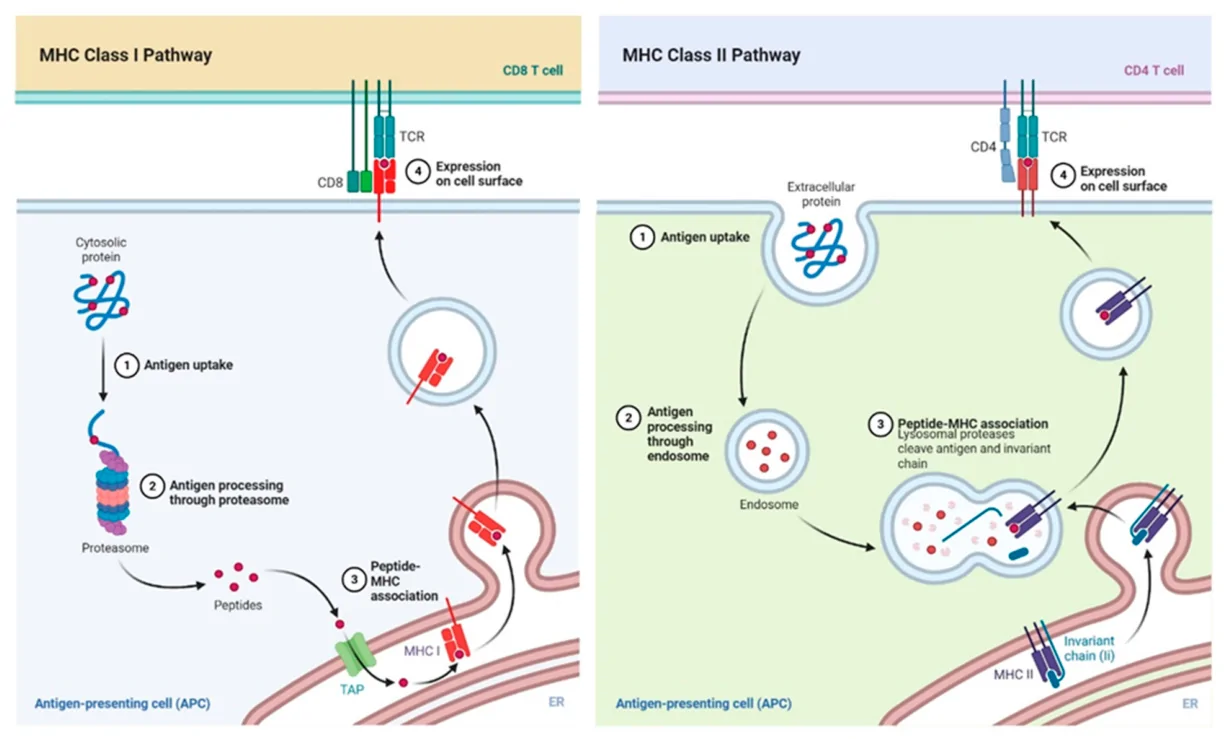
Antigen processing and presentation through MHC class I and class II pathways. Fungal antigens are processed and presented by antigen-presenting cells (APCs) through two major distinct pathways. In the MHC class I pathway, intracellular fungal proteins are degraded by the proteasome into peptide fragments. These peptides are transported into the endoplasmic reticulum (ER) through the transporter associated with antigen processing (TAP), where they are loaded onto MHC class I molecules. The resulting fungal peptide–MHC class I complexes are transported to the cell surface and recognized by T-cell receptors (TCRs) on CD8 T cells in association with the CD8 co-receptor, promoting antifungal cytotoxic T-cell responses. In the MHC class II pathway, extracellular fungal proteins are internalized by APCs through endocytosis and degraded into peptides within endosomal/lysosomal compartments. MHC class II molecules synthesized in the ER associate with the invariant chain (Ii), which directs them to endosomal compartments. Proteolytic processing removes the invariant chain, allowing fungal-derived peptides to bind MHC class II molecules. The resulting peptide–MHC class II complexes are transported to the cell surface and recognized by TCRs on CD4 T cells together with the CD4 co-receptor. These interactions promote CD4 T-cell activation and cytokine-mediated antifungal immune responses. Together, MHC class I and class II antigen presentation pathways enable coordinated CD8 and CD4 T-cell responses against fungal infections (created in BioRender https://BioRender.com/rwiesck).

Upon microbial infection, pathogen-derived proteins are proteolytically processed into peptide fragments that are subsequently presented by MHC molecules. In fungal infections, exogenous fungal antigens acquired through phagocytosis or endocytosis are primarily processed within the endosomal–lysosomal pathway and presented on MHC class II molecules, thereby activating CD4 lymphocytes. Additionally, professional antigen-presenting cells can process exogenous fungal materials via cross-presentation onto MHC class I molecules to activate CD8 lymphocytes. The incorporation of pathogen-derived peptides into self-MHC molecules converts the self-peptide–MHC into an altered-self ligand recognized by TCRs [24] (Figure 1). However, productive T-cell activation requires more than antigen recognition alone; full activation depends on the integration of co-stimulatory signals together with cytokine-mediated cues provided by professional APCs, ensuring acquired immune responses are tightly regulated to maximize host protection while minimizing immunopathogenesis [25,26].

**Figure 2:**
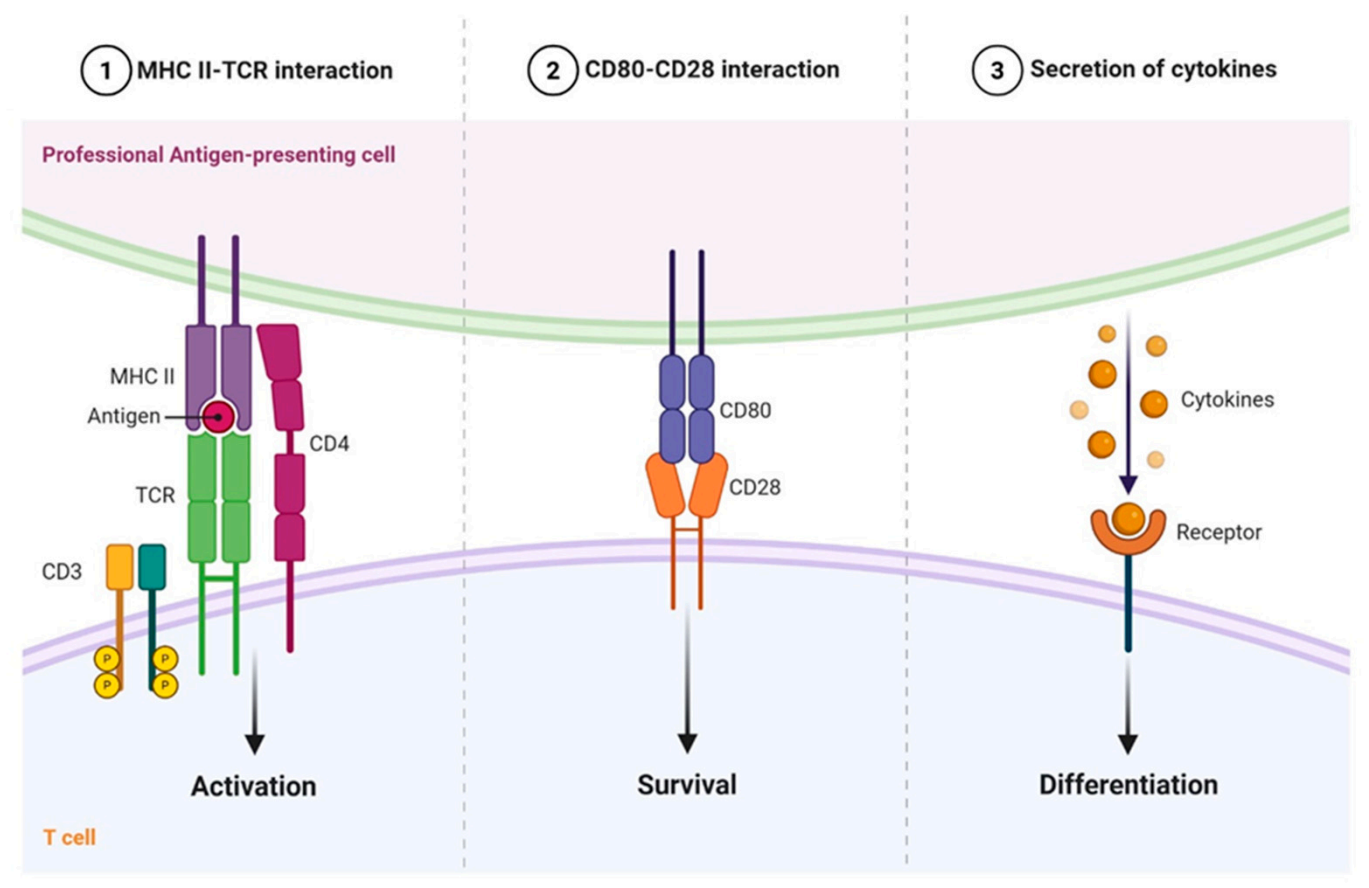
CD4 helper T-cell activation through antigen recognition, co-stimulation, and cytokine signaling. T-cell activation requires coordinated interactions between professional antigen-presenting cells (APCs) and T cells. (1) Antigen recognition: the T-cell receptor (TCR) recognizes antigenic peptide presented by MHC II on the APC, with CD4 acting as a co-receptor and CD3 mediating intracellular signal transduction, resulting in T-cell activation. (2) Co-stimulatory signaling: interaction between CD80 on the APC and CD28 on the T cell provides an essential co-stimulatory signal that promotes T-cell survival and sustained activation. (3) Cytokine signaling: cytokines produced by APCs and other immune cells bind to specific receptors on T cells and regulate their proliferation, differentiation, and functional polarization. Together, antigen-specific TCR signaling, CD80–CD28 co-stimulation, and cytokine-mediated signaling constitute the major signals required for effective T-cell activation and differentiation (created in BioRender https://BioRender.com/rwiesck).

Professional antigen-presenting cells are specialized immune cells that provide the essential link between innate and acquired immunity by capturing, processing, and presenting foreign antigens to T lymphocytes. Although virtually all nucleated cells can present endogenous peptides via MHC class I molecules to CD8 CTLs, professional APCs (pAPCs) are uniquely distinguished by their capacity to prime naïve T cells and initiate antigen-specific acquired immune responses. This function depends on the coordinated delivery of three complementary signals. First, pAPCs constitutively express MHC class II molecules, in addition to MHC class I, enabling them to internalize exogenous pathogens, process them into peptide fragments, and present these peptides on MHC class II molecules for recognition by CD4 helper T cells. Second, pAPCs provide indispensable co-stimulatory signals through surface molecules such as CD80 and CD86, which bind to CD28 on T cells. Because TCR recognition of peptide–MHC complexes alone is insufficient for full activation, the absence of these co-stimulatory signals results in T-cell anergy and immune tolerance. Third, activated pAPCs secrete polarizing cytokines that instruct naïve T cells to differentiate into specialized effector subsets, including TH1, TH2, TH17, and T follicular helper (TFH) cells, thereby tailoring the acquired immune response to the nature of the invading pathogen. The integration of these three signals ensures effective T-cell activation, functional specialization, and the generation of appropriate pathogen-specific immunity (Figure 2). Unlike pAPCs, non-professional APCs or amateur antigen-presenting cells (aAPCs)—including fibroblasts, endothelial cells, and certain epithelial cells—do not constitutively express MHC class II molecules or the co-stimulatory signals required for naïve T-cell activation. Under conditions of intense inflammation, particularly in response to cytokines such as IFN-γ, these cells can be induced to express MHC class II and present antigenic peptides. However, their antigen-presenting function is largely restricted to interacting with previously primed or activated T cells, as they generally lack the capacity to provide the co-stimulatory signals necessary to initiate primary immune responses by activating naïve T cells [25,27].

## 3. Mechanisms of Antigen Processing and Presentation

The cellular and molecular basis of MHC function relies on distinct intracellular antigen-processing pathways that generate fungal peptides and load them onto cell-surface MHC glycoproteins for presentation to T lymphocytes [28].

### 3.1. The Exogenous Antigen Processing Pathway and MHC Class II Restriction

Protective CD4 T-cell immunity against fungal pathogens depends predominantly on the exogenous antigen processing pathway. pAPCs, including DCs, macrophages, and B lymphocytes, internalize extracellular fungal structures such as spores, conidia, and hyphal fragments through phagocytosis or receptor-mediated endocytosis. Pattern recognition receptors (PRRs), including Dectin-1, which recognizes fungal β-glucan, and Toll-like receptors (TLRs), facilitate fungal antigen recognition and uptake while simultaneously delivering the maturation signals required for APC activation [29].

Following internalization into the endosomal–lysosomal compartments, fungal proteins are proteolytically degraded by acid-dependent proteases, primarily cathepsins, into peptide fragments typically ranging from 13 to 25 amino acids in length [30]. Concurrently, newly synthesized MHC class II molecules, assembled within the endoplasmic reticulum (ER) and stabilized by the invariant chain (commonly known as CD74), are transported to late endosomal compartments. Within these compartments, the invariant chain undergoes proteolytic degradation, leaving only the class II-associated invariant chain peptide (CLIP) occupying the peptide-binding groove. The non-classical MHC molecule HLA-DM subsequently catalyzes the exchange of CLIP for higher-affinity fungal peptides. Once loaded with antigenic peptides, the stable peptide–MHC class II complexes are transported to the plasma membrane, where they are surveyed by the TCRs expressed on CD4 T lymphocytes [31].

Evidence further indicates that human B lymphocytes can function as pAPCs capable of priming naïve T cells against C. albicans through an antibody-independent mechanism. This process requires coordinated signaling through MHC class II molecules together with the costimulatory molecules CD80 (B7-1) and CD86 (B7-2). Functional blockade of HLA-DR using specific monoclonal antibodies significantly attenuates T-cell activation, demonstrating the essential role of MHC class II-mediated antigen presentation in initiating antifungal acquired immune responses [32].

### 3.2. The Endogenous Pathway, Cross-Presentation, and MHC Class I Restriction

MHC class I molecules are constitutively expressed on nearly all nucleated cells and primarily present endogenous antigens, including proteins synthesized within the cell, to CD8 CTLs. In the canonical MHC class I antigen-processing pathway, cytosolic proteins are degraded by the proteasome into peptide fragments approximately 8–10 amino acids in length. These peptides are subsequently transported into the endoplasmic reticulum by the transporter associated with antigen processing (TAP) complex, where they are loaded onto nascent MHC class I molecules with the assistance of the peptide-loading complex, including molecular chaperones such as calnexin, calreticulin, and tapasin [33]. The resulting stable peptide–MHC class I complexes are then transported to the plasma membrane for recognition by CD8 T cells [31,34].

**Figure 3:**
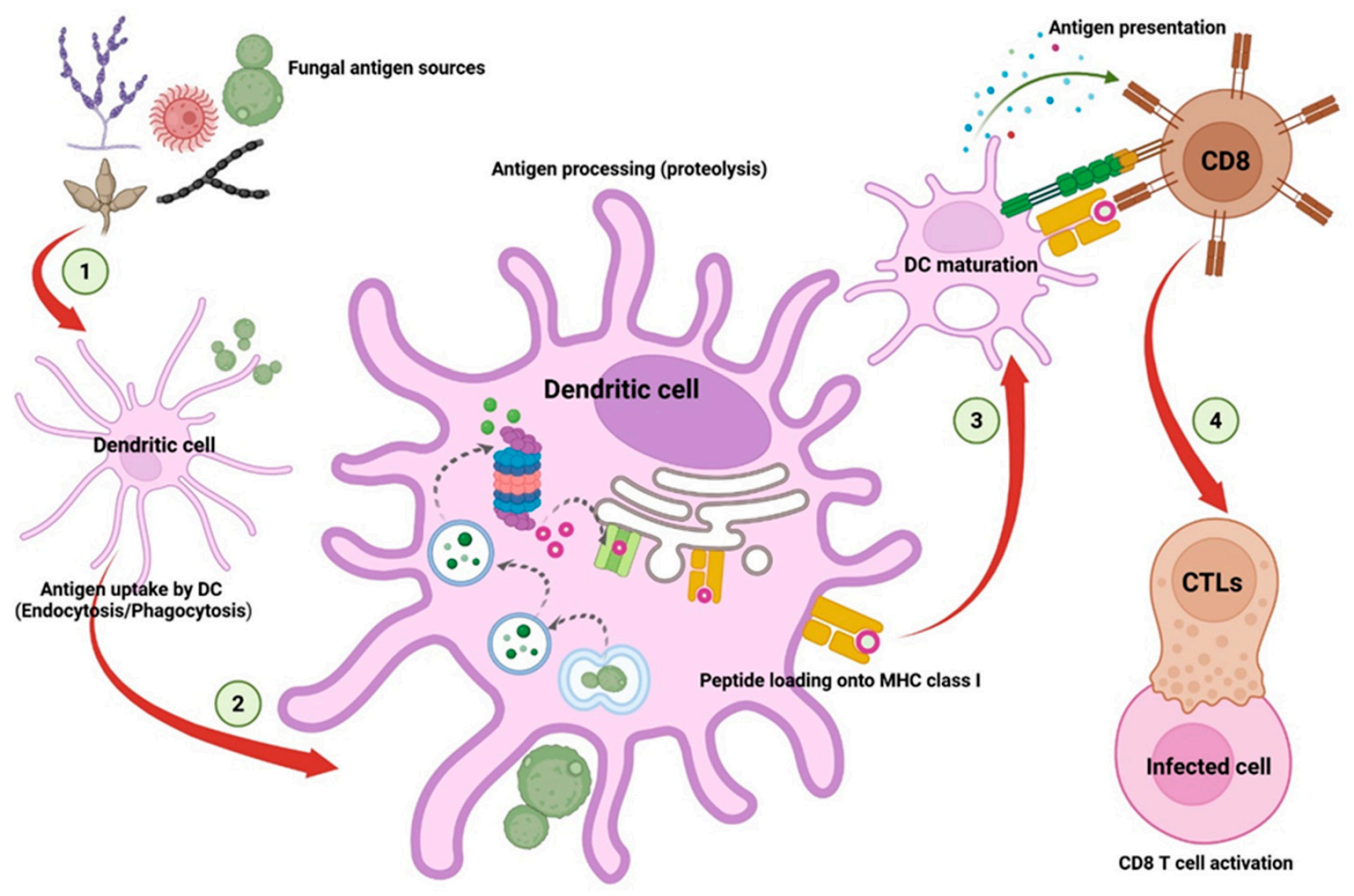
Proposed mechanism of fungal antigen cross-presentation by dendritic cells and subsequent activation of CD8 T cells. Fungal antigens derived from yeast, hyphae, and other fungal structures are captured by dendritic cells (DCs) through endocytosis and/or phagocytosis (1). These exogenous antigens are processed into peptides and delivered to the MHC class I loading machinery through cross-presentation (2). Fungal PAMPs promote DC activation and maturation, enhancing MHC and costimulatory molecule expression. Mature DCs migrate to lymphoid tissues, where fungal peptide–MHC class I complexes activate naïve or antigen-specific CD8 T cells, driving their clonal expansion and differentiation into CTLs (3). CTLs subsequently recognize and eliminate infected host cells presenting the corresponding fungal peptide–MHC class I complex (4). Thus, cross-presentation enables exogenous fungal antigens to induce MHC class I–restricted CD8 T-cell antifungal immunity, even without direct DC infection (created in BioRender https://BioRender.com/rwiesck).

Because most pathogenic fungi replicate either extracellularly or within phagosomal compartments of professional phagocytes, as exemplified by *B. dermatitidis*, *T. marneffei* and *H. capsulatum*, fungal antigens are generally classified as exogenous antigens [35,36,37]. Consequently, the generation of antifungal CD8 T-cell responses depends predominantly on the specialized process of cross-presentation, whereby extracellular fungal antigens are presented on MHC class I molecules. During this process, specialized subsets of conventional type 1 dendritic cells, characterized by the expression of CD8α, XCR1, and the transcription factor BATF3, internalize fungal antigens and reroute them from the endosomal–lysosomal compartment into the cytosol. Once translocated, these antigens enter the canonical MHC class I processing pathway, allowing peptide loading onto MHC class I molecules and subsequent activation of CD8 T cells [34,38]. Experimental evidence further demonstrates that, even in the absence of CD4 T-cell help, exogenous fungal antigens can efficiently stimulate memory CD8 T cells through MHC class I-restricted cross-presentation, resulting in long-lasting antifungal immunity mediated by the production of TNF-α, IFN-γ, and GM-CSF [13,38] (Figure 3).

Unfortunately, empirical laboratory evidence regarding fungal cross presentation remains limited and warrants further extensive investigation. Nevertheless, a particularly compelling study using Batf3-deficient mice, which lack conventional type 1 dendritic cells (cDC1s), demonstrated profound immunological consequences. For instance, during *Cryptococcus neoformans* infection, the absence of cDC1s leads to a significant reduction in IFN-γ secreting CD4 and CD8 T cells within the lungs and brain, resulting in a markedly increased fungal burden compared with wild-type mice. The loss of cDC1s also reduces the production of inducible nitric oxide synthase (iNOS) in microglia and inflammatory monocytes, demonstrating that cDC1s serve as a critical component in establishing a TH1 microenvironment for fungal clearance [39].

An investigation into dendritic cell dynamics following vaccination against *Blastomyces dermatitidis* revealed that the vaccine strongly activates migratory conventional cDC2s in draining lymph nodes. The cDC2 population and its response peaked on day 5 after vaccination, when cDC2s outnumbered all other dendritic cell subsets combined. Importantly, although cDC2s exhibited greater uptake of fungal immunogens, their average cross-presentation capacity was comparable to that of cDC1s. Furthermore, in an environment depleted of CD4 T cells, a counterintuitive phenomenon was observed: the absence of CD4 T cells unexpectedly enhanced dendritic cell activation, antigen uptake, expression of costimulatory molecules, and cross-presentation capacity, including that of cDC2s. This CD4-independent cDC2 response offers a potentially important new perspective for vaccine design in immunocompromised patients who lack sufficient CD4 T cell assistance to mount an adequate immune response [40].

Perhaps one of the most critical insights into immunological memory networks is the discovery of T cell cross-reactivity. TCRs that recognize a peptide from one fungal species may possess sufficient structural flexibility to interact with shared epitope sequences from other fungal species. In vitro tissue culture experiments have confirmed that TH1 memory cells generated following *A. fumigatus* infection exhibit robust cross-reactivity against immunogens from *C. albicans*, *Penicillium* sp., and *Scedosporium* sp., while also providing effective protection against Mucorales infections. This cross-reactivity phenomenon, including cross-kingdom reactivity directed against bacteria such as Staphylococcus aureus, has inspired the development of pan-fungal vaccines that use a single shared antigen to provide broad-spectrum protection [41,42].

## 4. Non-Classical Immune Surveillance: MR1-Restricted MAIT Cell Responses to Fungal Secondary Metabolites

**Figure 4:**
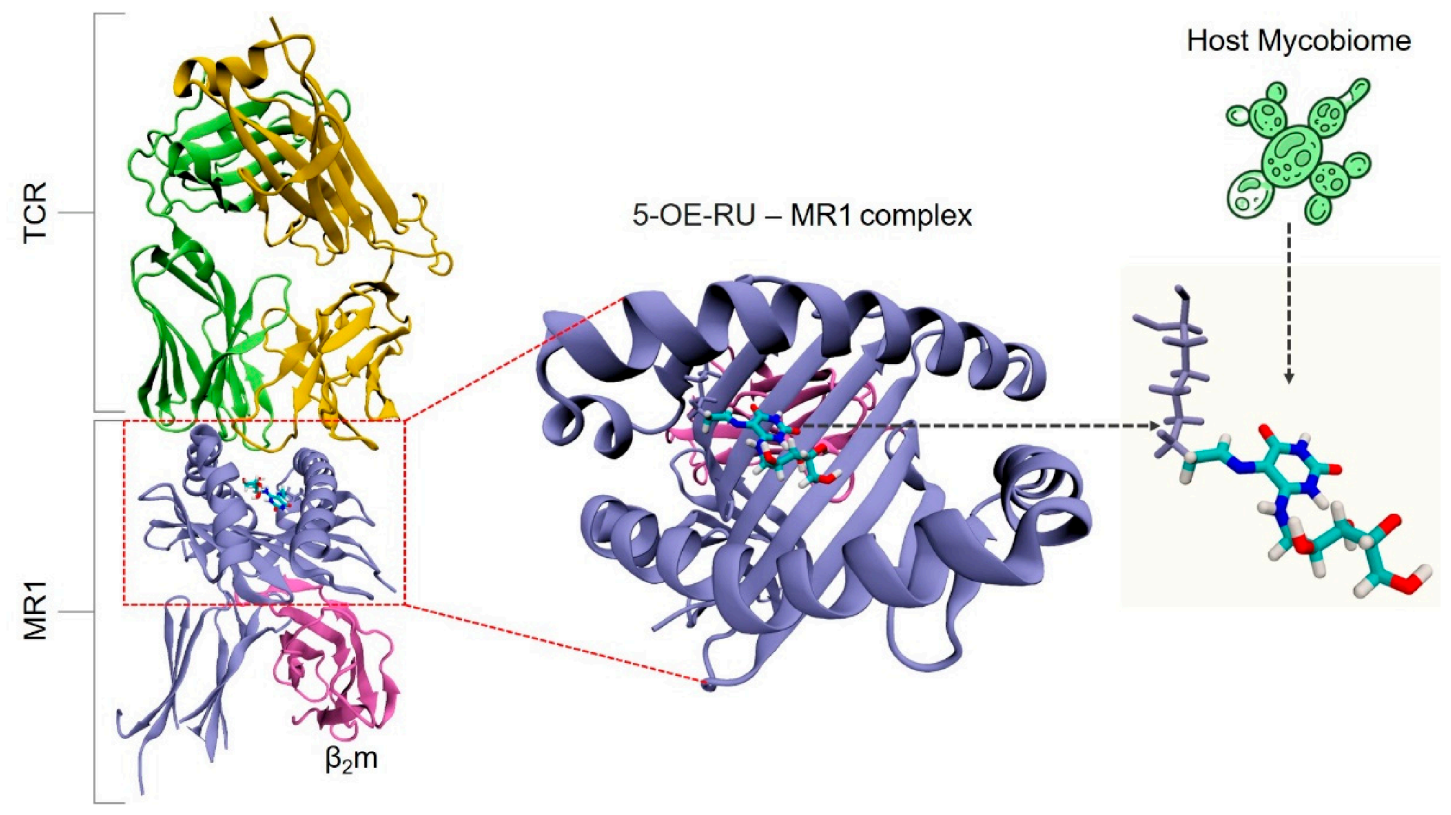
Crystal structure of MAIT TCR-MR1 complex interacting with 5-OE-RU. (PDB ID: 4NQE). The structural representation highlights the relationship among the microbial source (including human mycobiome) of the riboflavin-derived metabolite, the chemical structure of 5-OE-RU, its accommodation and covalent stabilization within MR1, and subsequent recognition by the TCR. TCR is shown as green and gold ribbons, positioned above the MR1 molecule. MR1 consists of the heavy chain (light purple) associated with β2-microglobulin (magenta). The boxed region provides an enlarged view of the MR1 antigen-binding groove and the bound 5-OE-RU molecule. The antigen-binding site is formed by two α-helices positioned over a β-sheet platform, creating a predominantly hydrophobic cavity that accommodates the ligand. The accompanying schematic illustrates the microbial origin of 5-OE-RU, a reactive metabolite generated by microorganisms within the host, thereby providing a molecular link between fungal metabolism and host immune surveillance. In the magnified three-dimensional ball-and-stick model of the ligand bound within the antigen-binding site, atom colors follow standard chemical conventions: Carbon atoms are shown in cyan, Nitrogen atoms in blue, Oxygen atoms in red, and Hydrogen atoms in white. The solid purple stick structure represents the carbon backbone and nitrogen atom of the MHC-K43 side chain from the MR1, which serves as the anchoring site for the ligand within the MHC binding groove (created in VMD visualization program; https://www.ks.uiuc.edu/Research/vmd/ (accessed on 22 October 2026)).

Beyond the classical MHC class I and class II antigen presentation pathways, immune surveillance also involves non-classical MHC molecules mediated by specialized antigen-presenting molecules. Among these, MHC class I-related protein 1 (MR1) is a highly conserved, monomorphic antigen-presenting molecule that binds metabolites derived from the riboflavin (vitamin B2) biosynthetic pathway, which are produced by many bacteria, commensal microorganisms, and fungi, before presenting them to mucosal-associated invariant T (MAIT) cells [43,44]. The evolutionary conservation of the riboflavin biosynthetic pathway across diverse fungal genera, including Saccharomyces, Aspergillus, Mucorales and Candida species, as well as numerous bacterial species, provides a universal molecular signature for MR1-dependent immune surveillance [45]. In contrast, mammalian host cells lack the enzymatic machinery required for riboflavin biosynthesis; therefore, these metabolites represent unambiguous indicators of microbial invasion rather than host-derived molecules [46]. Among the MR1 ligands identified to date, the most potent activators of MAIT cells are unstable pyrimidine-based intermediates generated during microbial riboflavin biosynthesis, particularly 5-(2-oxopropylideneamino)-6-D-ribitylaminouracil (5-OP-RU) and the related ligand 5-OE-RU [46] (Figure 4). These highly reactive metabolites form covalent Schiff-base complexes with MR1, stabilizing the molecule and enabling efficient recognition by the semi-invariant T-cell receptor expressed on canonical MAIT cells. The remarkable sensitivity of MAIT cells to these ligands allows rapid detection of microbial infection at mucosal surfaces and promotes early secretion of effector cytokines, including IFN-γ, TNF-α, and IL-17, thereby bridging innate and acquired immunity [47].

**Figure 5:**
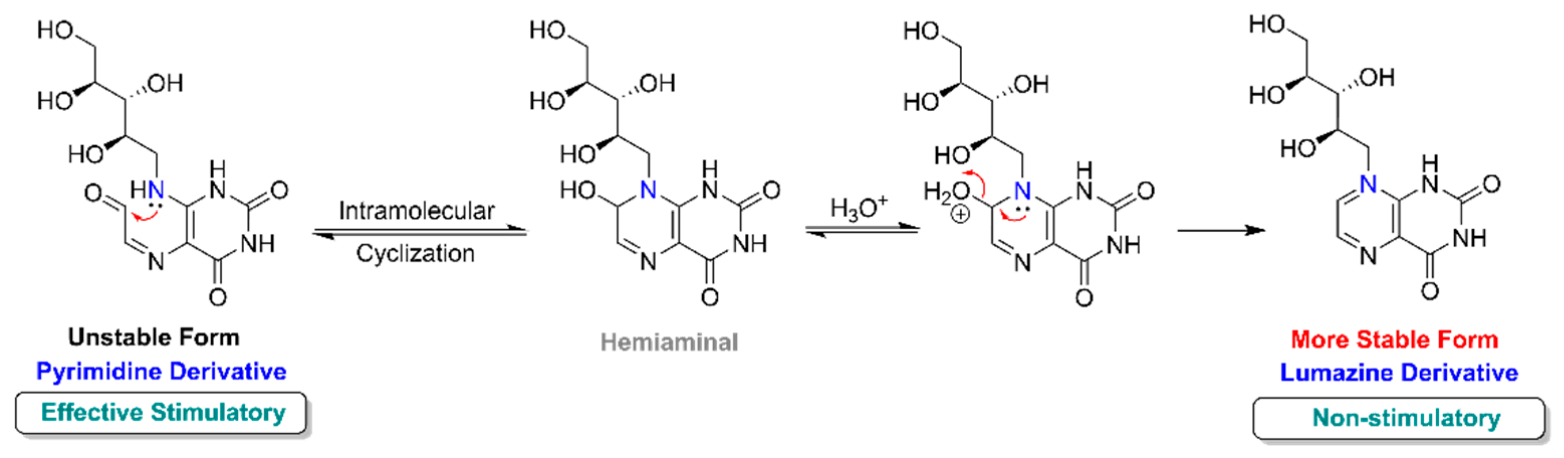
Proposed mechanism for conversion of the stimulatory pyrimidine intermediate into the non-stimulatory lumazine derivative. The open-chain, ribityl-substituted aminouracil undergoes intramolecular cyclization, in which the exocyclic ribitylamino nitrogen atom attacks the carbonyl carbon, to generate a cyclic hemiaminal intermediate. Under physiological conditions, protonation of the hydroxyl group promotes loss of water, driving aromatization of the pyrazine ring and yielding the thermodynamically more stable, fully conjugated lumazine derivative. This reaction converts the immunologically active pyrimidine species, which is capable of efficiently binding to MR1, into an inactive, non-stimulatory lumazine product, illustrating a chemical basis for the limited stability of this immunogenic ligand.

Based on this, it rapidly forms the more stable bicyclic lumazine structure through intramolecular dehydration and ring closure. Analysis of the crystal structure of the ternary complex comprising the MAIT TCR, MR1, and the antigen 5-OE-RU (PDB ID: 4NQE), which provides atomic-level information at a resolution of 2.10 Å, a key mechanistic feature is revealed that the pyrimidine adduct 5-OE-RU can form a reversible covalent Schiff-base bond with lysine at position 43 (Lys43), which is deeply located within the binding groove of MR1. Chemically, 5-OE-RU consists of the uracil ring, a ribityl moiety, and a highly reactive aliphatic tail containing an α-iminocarbonyl functionality. Under physiological conditions, 5-OE-RU is highly unstable. The reaction occurs when the -NH2 reacts with C=O of the aldehyde to form a hemiaminal, followed by dehydration to yield an aromatic ring (Figure 5). Moreover, the half-life of these pyrimidine intermediates in aqueous environments is extremely short because of their susceptibility to autoxidation and the kinetics of intramolecular ring closure [48]. Therefore, for 5-OE-RU to function as an effective stimulatory ligand, it must be captured and chemically stabilized by an external protein before irreversible ring closure occurs. MR1 thus functions as an exceptionally effective chemical trap for this process [49,50].

**Figure 6:**
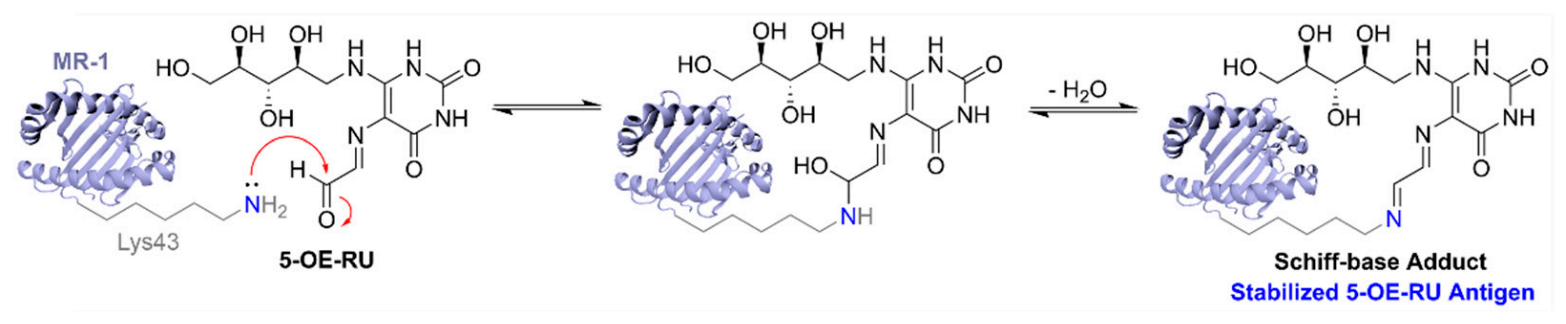
Mechanism of covalent capture and stabilization of the labile pyrimidine antigen 5-OE-RU by MR1 through Schiff-base formation with Lys43. The amino group of Lys43 in the MR1 antigen-binding groove undergoes nucleophilic attack on the reactive aldehyde carbon of 5-OE-RU (left), forming a hemiaminal intermediate in which the lysine nitrogen is covalently linked to the ligand. Loss of water leads to the formation of the stable Schiff-base adduct, generating 5-OE-RU covalently bound to MR1 (right). This covalent trapping locks the otherwise unstable precursor into a stabilized antigenic conformation within the MR1 binding groove, enabling durable presentation to the MAIT cell.

Under physiological pH, the amino group of an exposed lysine residue typically has a relatively high pKa (approximately 10.5) and predominantly exists in its protonated form (-NH3^+^), rendering it a relatively poor nucleophile. However, the unique microenvironment within the A′-pocket of MR1 dramatically alters the chemical reactivity of Lys43. The water-depleted and hydrophobic environment of this pocket modifies the local electrostatic environment, resulting in a substantial reduction in the effective pKa of Lys43. Consequently, a fraction of the Lys43 side chains exists in the deprotonated state and exhibits strong nucleophilic character (-NH2). The reaction proceeds through nucleophilic attack by the primary amino group of Lys43 on the carbonyl carbon of 5-OE-RU, generating an unstable hemiaminal intermediate. This intermediate rapidly undergoes dehydration to form a stable carbon–nitrogen double bond (-C=N-), producing the Schiff-base adduct (Figure 6). Although this covalent linkage is reversible, it acts as a strong thermodynamic sink. The Schiff base serves as an anchoring point for the aliphatic tail of 5-OE-RU, restricting its rotational degrees of freedom and thereby enhancing the overall stability of the protein–ligand complex, including its resistance to thermal denaturation. Importantly, the formation of the 1,2-diimino moiety physically locks the ligand in a configuration that prevents intramolecular cyclization. This mechanism has been clearly demonstrated through mutagenesis studies. Recombinant MR1 carrying a Lys43-to-Ala43 substitution (MR1-K43A) retains an intact aromatic binding pocket but lacks the nucleophilic amino group required for Schiff-base formation. Although MR1-K43A can transiently accommodate 5-OE-RU through non-covalent interactions, it lacks the capacity to efficiently trap and stabilize the ligand, fails to confer sufficient stability to the resulting protein–ligand complex, and consequently loses the ability to effectively promote antigen presentation at the cell surface and activate MAIT cells. Thus, Schiff-base formation is essential for the structural stability and immunological activity of 5-OE-RU antigen presentation [51]. Furthermore, recent studies have identified additional populations of MR1-restricted T cells that do not express the canonical TRAV1-2 T-cell receptor α-chain (TRAV1-2-negative MR1-restricted T cells). Unlike conventional MAIT cells, these non-canonical MR1-restricted T cells recognize a much broader repertoire of microbial metabolites and non-peptide antigens, indicating an unexpectedly diverse MR1-dependent antigen recognition system that extends well beyond the classical paradigm of peptide-mediated MHC binding [52].

## 5. Experimental Approaches to Assess MHC Function

The precise identification of immunodominant fungal epitopes and the MHC alleles that restrict their presentation requires a combination of complementary experimental approaches. The integration of high-throughput peptide screening, functional cellular assays, and advanced flow cytometric techniques constitutes the cornerstone of current experimental evidence in antifungal immunology [53,54].

### 5.1. Overlapping Peptide Libraries and Matrix Mapping

To comprehensively interrogate the fungal proteome or specific full-length fungal antigens, investigators commonly employ synthetic overlapping peptide libraries. The most widely used strategy is to synthesize the entire antigen sequence as a series of 15-amino-acid peptides (15-mers or pentadecapeptides), with each consecutive peptide overlapping the preceding peptide by 11 amino acids [55]. The choice of peptide length and overlap is strategically important for several reasons:

I. Optimal length for MHC class II presentation. Fifteen-mer peptides are ideally suited for presentation by MHC class II molecules, whose peptide-binding grooves are open at both ends and can accommodate longer peptide ligands.

II. Compatibility with MHC class I presentation. Although MHC class I molecules preferentially bind peptides of approximately 8–10 amino acids, exogenously added 15-mer peptides can undergo extracellular trimming by serum peptidases, generating shorter peptide fragments capable of fitting into the closed-ended binding groove of MHC class I molecules.

Consequently, these peptides can also stimulate CD8 T-cell responses in assays such as ELISpot. An overlap of 11 amino acids ensures that every potential naturally processed 8–12-mer epitope generated by APCs is represented in its entirety within at least one synthetic peptide in the screening library. To efficiently identify immunogenic epitopes, overlapping peptide libraries are frequently organized into a two-dimensional matrix format, in which peptides are pooled according to rows and columns. A T-cell response to one row pool and one column pool allows the immunogenic peptide to be rapidly identified as the peptide located at the intersection of the two positive pools. This matrix mapping strategy substantially reduces the number of individual peptides that must be tested while maintaining high-resolution epitope identification [56,57].

### 5.2. Enzyme-Linked ImmunoSpot (ELISpot) Assay and Intracellular Cytokine Staining

The enzyme-linked immunospot (ELISpot) assay is a highly sensitive technique for quantifying the frequency of antigen-specific T cells that secrete particular cytokines at the single-cell level. In antifungal immunology, ELISpot has been widely employed to measure the production of interferon-γ (IFN-γ), a hallmark of TH1 and CD8 T-cell responses; interleukin-17 (IL-17), indicative of TH17 immunity; and interleukin-4 (IL-4), which reflects TH2 polarization and allergic immune responses [58,59]. For example, in studies examining immune responses of healthy individuals to antigens derived from the filamentous fungus A. fumigatus (Asp f proteins), ELISpot has demonstrated robust IFN-γ production in response to recombinant fungal antigens. These findings enable the discrimination of protective antifungal cellular immunity, characterized by TH1-dominant responses, from allergic immune responses, which are typically associated with TH2 polarization and contribute to respiratory complications such as allergic bronchopulmonary aspergillosis [60]. Intracellular cytokine staining (ICS), when combined with multiparameter flow cytometry, provides functional and phenotypic characterization of antigen-specific T-cell responses at the single-cell level. By treating antigen-stimulated cells with protein transport inhibitors, such as Brefeldin A or Monensin, newly synthesized cytokines are retained intracellularly rather than being secreted into the extracellular milieu. Following cell fixation and permeabilization, intracellular cytokines can be detected using fluorochrome-conjugated antibodies. This approach enables investigators to precisely identify the cellular subsets responsible for cytokine production in response to fungal epitopes. For example, ICS can distinguish cytokine-producing CD4 helper T cells from CD8 CTLs, while simultaneously characterizing their differentiation and memory phenotypes through the expression of markers such as CD44, CD62L, CCR7, or CD45RO, depending on the experimental system [61].

Recently, a novel host-based diagnostic strategy utilizing IFN-γ release assays (IGRAs) successfully differentiates between naïve individuals and those exposed to *T. marneffei*, offering a critical pathway for detecting clinically silent, latent infections in vulnerable populations. *T. marneffei* is an endemic fungus in Southeast Asia and represents a leading cause of mortality among patients with advanced HIV infection [62]. Conventional pathogen-based diagnostics are limited by their inability to detect the fungus until the disease reaches advanced stages, when antifungal treatment is least effective. Deploying host-based IGRAs addresses this clinical gap by identifying latent infections early, which enables clinicians to initiate prophylactic therapy and prevent progression to active, life-threatening disease. The immunological mechanism relies on host memory T cells that recall prior *T. marneffei* exposure. When stimulated by antigen-presenting cells introducing the *T. marneffei* specific recombinant mannoprotein Mp1p (rMp1p), these memory T cells mount a robust immune response characterized by the secretion of IFN-γ. Quantifying this elevated cytokine release during subsequent laboratory exposure allows for clear differentiation between exposed individuals (those with latent or active infections) and naïve individuals [63]. A similar diagnostic assay has been developed for histoplasmosis [64].

### 5.3. Proliferation and Functional Blocking Assays

Lymphocyte proliferation assays provide a robust means of quantifying the clonal expansion of T cells following the recognition of cognate peptide–MHC complexes. These assays are commonly based on the incorporation of radioactive nucleotides, such as 3H-thymidine, into newly synthesized DNA or on the progressive dilution of fluorescent cell-tracing dyes, including carboxyfluorescein succinimidyl ester (CFSE), whose fluorescence intensity decreases by approximately one-half with each successive round of cell division [32].

To unequivocally establish MHC function, proliferation assays are frequently combined with monoclonal antibody blocking assays. In these experiments, APCs are first pulsed with fungal antigens and subsequently co-cultured with antigen-specific T cells. Monoclonal antibodies directed against specific MHC molecules, such as anti-HLA-DR, anti-HLA-DP, anti-HLA-DQ, or anti-HLA class I, are then added to the culture system. Complete inhibition of T-cell proliferation and cytokine secretion following treatment with an anti-HLA-DR antibody provides compelling evidence that the fungal epitope is presented exclusively in the context of HLA-DR-restricted antigen presentation [42,65].

In severe or disseminated fungal infections, prolonged exposure to fungal antigens presented on MHC molecules frequently induces T-cell exhaustion, characterized by increased expression of inhibitory immune checkpoint receptors such as programmed cell death protein 1 (PD-1). Functional studies have demonstrated that blockade of the PD-1 pathway using PD-1-specific monoclonal antibodies restores the proliferative capacity and effector cytokine production of fungus-specific T cells, indicating immune checkpoint blockade as a promising immunotherapeutic strategy for invasive fungal diseases [66,67].

### 5.4. Peptide–MHC Multimer/Tetramer Technology

The most direct physical evidence supporting MHC function is provided by peptide–MHC multimer technology, most commonly in the form of peptide–MHC tetramers. Recombinant MHC molecules, such as human HLA-DR4 or HLA-A*02, are refolded in vitro in the presence of a defined fungal epitope peptide. The resulting peptide–MHC monomers are subsequently biotinylated and assembled into tetramers using fluorophore-conjugated streptavidin, generating highly specific reagents for the identification of antigen-specific T cells [12,68].

Because the affinity of a single TCR for an individual peptide–MHC is inherently low, tetramers rely on multivalent binding to achieve high overall avidity, thereby enabling stable binding to antigen-specific T cells [69]. When combined with multiparameter flow cytometry, peptide–MHC tetramers permit the direct visualization and quantitative analysis of the frequency, phenotype, activation status, and differentiation profile of fungus-specific T cells in vivo without requiring indirect functional assays. This technology has become indispensable for monitoring the expansion of antigen-specific CD4 T cells following vaccination with dimorphic fungal antigens and for characterizing acquired antifungal immune responses with high sensitivity and specificity [70].

To facilitate the translation of epitope discoveries into clinically relevant human immune responses without conducting early-stage human studies, investigators have developed humanized transgenic mouse models expressing human MHC molecules. A well-established example is the HLA-DR4 (DRB1*0401) transgenic mouse generated on the C57BL/6 genetic background. These mice are genetically manipulated to lack endogenous murine MHC class II molecules (I-A and I-E) while expressing human HLA-DR4 as the sole class II antigen-presenting molecule [12]. Vaccination followed by challenge with virulent fungal conidia in these models provides direct in vivo evidence that protective antifungal immunity can be mediated through HLA-restricted antigen presentation, thereby validating fungal vaccine candidates and human MHC-restricted T-cell epitopes for translational immunological research [71].

### 5.5. MHC Blocking Assays

MHC blocking assays provide functional evidence for MHC function by using monoclonal antibodies that bind to MHC molecules and interfere with antigen recognition by T cells [72,73]. A reduction in T cell activation, assessed by cytokine production, cell proliferation, or cytotoxicity, indicates that the targeted MHC molecules contribute to antigen presentation.

HLA genes are highly polymorphic and include the classical class I loci HLA-A, HLA -B, and HLA-C, as well as the class II loci HLA-DR, HLA-DP, and HLA-DQ. Blocking antibodies differ in their specificity, with some recognizing multiple HLA molecules within a class and others targeting particular loci. Selecting appropriate antibodies is therefore important when investigating the pathways involved in fungal antigen recognition. In a typical assay, APCs are exposed to fungal antigens and co-cultured with the T cell population of interest. Blocking antibodies are added to interfere with the interaction between TCRs and the relevant pMHC complexes. If T cell activation decreases following blockade, this supports a role for the targeted HLA molecule in the response. Conversely, little or no reduction suggests that other presentation pathways may contribute, although incomplete blockade and other technical factors must first be excluded.

Several limitations should be considered when interpreting these assays. Incomplete inhibition may occur when antibody binding is insufficient, when pMHC complexes are abundant on the APC surface, or when the antibody does not effectively block the relevant interaction. Differences in HLA alleles may also affect antibody recognition and reduce the effectiveness of particular antibody clones. Residual T cell activation should therefore not automatically be interpreted as evidence of an MHC-independent response.

Using combinations of blocking antibodies may improve coverage when individual clones do not recognize all relevant HLA molecules. For example, combining antibodies with complementary specificities against HLA-DR and HLA-DQ, together with broader class II blocking reagents, may provide more effective inhibition than a single antibody alone. The suitability of combinations involving L243, WR18, and SPV-L3 should be established for the experimental system, including their specificity, compatibility, and ability to block the HLA alleles expressed by the APCs [74]. Failure of conventional HLA class I and class II antibodies to inhibit a fungal antigen-specific T cell response may also prompt investigation of alternative antigen presentation pathways. For example, if responses by CD4 or double-negative CD4 and CD8 T cells to fungal lysates persist despite treatment with W6/32, L243, SPV L3, and B7/21, additional experiments are needed to determine the mechanism involved. One possibility is recognition through nonclassical antigen presentation molecules, including MR1. MR1 presents certain microbial-derived metabolites to MAITs, and riboflavin biosynthesis products from fungi may provide relevant ligands in this context. However, the persistence of T-cell responses despite conventional HLA blockade does not necessarily indicate MR1-dependent recognition or implicate MAITs. These possibilities require further investigation using MR1-specific blocking antibodies, appropriate cellular phenotyping, and complementary experimental approaches. Such analyses can help differentiate conventional MHC-restricted antigen recognition from alternative pathways that contribute to T-cell responses against fungal antigens.

## 6. Empirical Evidence of MHC Function in Clinically Important Fungal Antigens

The application of the experimental approaches described above has led to the successful identification of numerous immunodominant fungal antigens that are recognized in a highly MHC-restricted manner. These studies have provided compelling evidence that antigen-specific T-cell responses against fungal pathogens are critically dependent on the presentation of defined peptide epitopes by particular MHC molecules. Such findings have significantly advanced our understanding of acquired immunity while providing a rational foundation for epitope-based vaccine development, immunodiagnostic assays, and T-cell–directed immunotherapies. To date, MHC-restricted immunodominant epitopes have been identified across a broad spectrum of clinically important fungal pathogens, as discussed in the following sections [75,76]. Table 1 summarizes characterized fungal antigenic epitopes derived from major human fungal pathogens, highlighting their associated antigens, peptide sequences, MHC restriction elements, and immunodominant T-cell responses, predominantly involving CD4 TH1/TH17 cells and CD8 cytotoxic T lymphocytes.

### 6.1. Aspergillus fumigatus: Asp f16 Antigen

Although CD4 T cells orchestrate and regulate antifungal immune responses, certain fungal antigens are also highly effective at eliciting MHC class I-restricted CD8 CTL responses. A. fumigatus allergen Asp f16, a glycosylhydrolase enzyme, represents one of the best-characterized examples [77,78]. Using a library of 104 overlapping 15-mer peptides spanning the entire 427 amino acid sequence of Asp f16, investigators pulsed mature fast DCs with individual peptides and co-cultured them with autologous peripheral blood mononuclear cells (PBMCs). Functional analyses demonstrated robust TH1 proliferation, while repeated in vitro priming generated antigen-specific CTLs with high functional avidity.

Matrix-based epitope mapping identified the 9-mer peptide WSIDGAVVR as the immunodominant epitope. Functional characterization revealed that this single epitope stimulated IFN-γ production and cytotoxic activity through dual antigen presentation pathways: HLA- DRB1-0301-restricted CD4 T-cell activation and HLA-B*3501-restricted CD8 T-cell activation via cross-presentation. Additional HLA class I-restricted epitopes identified within Asp f16 included YFKYTAAAL, LPLCSAQTW, and GTRFPQTPM, demonstrating that a single fungal protein can be processed into multiple peptides for presentation by diverse MHC class I molecules. Importantly, CTLs generated against these peptide–MHC complexes directly lysed Aspergillus hyphae and inhibited conidial growth, providing compelling evidence that MHC class I-restricted CD8 T cells contribute directly to antifungal protection [77].

### 6.2. Aspergillus fumigatus and Candida albicans: Crf1 Antigen

*A. fumigatus* and *C. albicans* are among the leading causes of life-threatening invasive fungal infections in immunocompromised individuals. The discovery of T-cell-mediated cross-protective immunity between these phylogenetically distant fungi represents a major advance in antifungal immunology. Central to this phenomenon is Crf1 (also designated Crh5), a cell wall-associated glucanase [79,80]. Epitope mapping using overlapping 15-mer peptide libraries identified Crf1-derived peptides capable of inducing robust CD4 TH1 memory responses. Importantly, these epitopes are presented by several common human HLA class II alleles, providing broad population coverage and the capacity to bind at least three HLA class II molecules that collectively represent more than 50% of the Caucasian population.

The Crf1 system illustrates how structural conservation can generate functional T-cell cross-reactivity. For example, the 15-mer epitope encompassing residues 162–176 (HTYTIDWNKDQTTWS) from *Aspergillus flavus* exhibits striking similarity to the homologous Crh1 sequence in *C. albicans* (HTYVIDWTKDAVTWS). T-cell clones expanded against the Aspergillus Crf1-derived p41 epitope vigorously recognized the corresponding Candida peptide through degenerate TCR recognition of structurally conserved peptide–MHC II complexes. In BALB/c mice, immunization with rCrf1 conferred cross-protective immunity against lethal *C. albicans* infection, demonstrating that MHC class II-restricted TH1 responses can be exploited to generate broad-spectrum antifungal immunity. Furthermore, a splice variant designated Crf2 contains a unique peptide (NSSAEPTAAVLAF) absent from Crf1 and Asp f9, providing a promising candidate for improving diagnostic specificity [79,81].

### 6.3. The Dimorphic Fungi: Endoglucanase 2 (Eng2)

Thermally dimorphic endemic fungi, including *B. dermatitidis*, *C. posadasii*, and *H. capsulatum*, are major causes of systemic mycoses in both immunocompetent and immunocompromised individuals across the Americas [82]. Endoglucanase 2 (Eng2) has been identified as a highly immunogenic glycoprotein localized to the fungal cell wall and surface. Despite sharing 50–68% sequence identity among homologues, immunization with Blastomyces Eng2 (Bl-Eng2) failed to confer cross-protection against Coccidioides Eng2 (Cp-Eng2) or Histoplasma Eng2 (Hc-Eng2), revealing the limitations of sequence homology as a predictor of protective immunity [12,83].

To investigate this lack of cross-protection, investigators performed comprehensive peptide-walking analyses to identify MHC class II-restricted epitopes. In Cp-Eng2, the dominant IFN-γ inducing peptide was the 13-mer TVWFFPRGNIPDD, whereas Hc-Eng2 elicited responses primarily against NFFNGPDPSNGYV and SSWARPIAHFTGC. Species-specific MHC restriction was confirmed using peptide–MHC II tetramers to track antigen-specific CD4 T cells following vaccination and fungal challenge. Flow cytometric analysis revealed extensive clonal expansion of tetramer-positive memory CD4 T cells in the lungs, with approximately 12% of activated CD44+ high CD4+ T cells specific for the immunodominant epitopes. These findings clearly demonstrate that, despite overall structural conservation, the location and identity of immunodominant epitopes differ substantially among Eng2 homologues, resulting in species-specific MHC function and incomplete cross-protective immunity [12].

**Table 1:** Currently characterized immunodominant fungal antigens and their MHC function, T-cell responses, validation methods, protective efficacy, and cross-protective profiles.

Pathogenic Fungus [Reference]	Antigen Nomenclature	Identified Epitope(s)/Peptide Sequence(s)	MHC Composition and Function	Dominant T-Cell Response	Validation Methods	Protective Efficacy	Specificity and Cross-Protection Profile
*Aspergillus fumigatus*/ *Candida albicans* [79]	Crf1 (Crh5)	p41/HTYTIDWNKDQTTWS (The peptide sequences of *A. fumigatus* Crf1/p41 and *C. albicans* Crh1/p41 differ by only one amino acid, with Thr replaced by Val at position 4.)	Broadly diverse HLA class II alleles.	CD4 TH1 (IFN-γ), with cross-reactivity.	Clinical-scale human PBMC expansion; T-cell adoptive-transfer models; tetramer staining.	Protection against lethal pulmonary challenge; expansion of CD4 TH1 memory cells; clearance of invasive fungal hyphae.	**- Major cross-species reactivity:** Pronounced cross-reactivity and cross-protection occur between *A. fumigatus* and *C. albicans* through the Crf1/p41 and Crh1/p41 epitopes. **-Specificity for other species:** Crf1/p41-specific T cells exhibit high specificity and do not respond to other Aspergillus species (*A. nidulans*, *A. niger*, and *A. terreus*) or other Candida species (*C. glabrata* and *C. krusei*). Only *A. flavus* exhibits slight cross-reactivity in certain T-cell clones, owing to its higher amino acid sequence similarity than that of the other species.
*Aspergillus fumigatus* [77]	Asp f16	YFKYTAAAL, LPLCSAQTW, GTRFPQTPM	HLA-B*3501 and HLA-DRB1*0301	CD8 CTL and CD4 TH1	- **^51^Cr-release Assay:** Measures cytotoxicity against autologous/HLA-matched BLCL and dendritic cells pulsed with peptides or fungal extracts. **- Flow Cytometry/Intracellular Cytokine Staining:** Measures the production of IFN-γ and IL-4, as well as the expression of CD69 and CD107a.	**-Conidial Killing:** The CTL culture supernatant contains secreted factors that can kill viable Aspergillus conidia and inhibit their metabolism. **- Hyphal Killing:** CTLs can directly kill fungal hyphae, and stimulation with the Asp f16 peptide pool further enhances their killing efficiency.	**- Amino Acid Sequence-Level Specificity:** According to the Protein Information Resource database, all three peptide sequences (YFK, LPL, and GTR) are uniquely found in *A. fumigatus*. **- MHC Subtype Cross-Restriction:** Cross-reactivity occurs exclusively with HLA-B3503 but not with HLA-B3502 or HLA-B*3508. This can be explained by differences in amino acids at positions 116 and 156 within the peptide-binding groove and at sites involved in TCR interactions.
*Coccidioides posadasii/C. immitis* [71]	rCpa1 (multivalent recombinant antigen)	Pep1-P1, Amn1-P9, Plb-P6 (TPLVVYIPNYPYTTWSNIST)	- MHC Specificity: All five epitope peptides were selected based on computational predictions indicating high-affinity and promiscuous binding to human MHC-II molecules, particularly HLA-DR4 (HLA-DRB1*0401).	CD4 TH1/TH17	HLA-DR4 transgenic mouse model; ELISPOT assays (IFN-γ/IL-17); formulation with glucan-chitin particles (GCPs) **- MHC function:** - In HLA-DR4 transgenic mice (with the endogenous mouse MHC-II genes knocked out), splenocytes respond to the human epitope peptides P1, P2, and P10, as well as the proteins rAg2/Pra and rPmp1. - In C57BL/6 mice (H2-I-Aᵇ haplotype), splenocytes respond only to the protein components (rAg2/Pra, Cs-Ag, and rPmp1) but not to any of the five human epitope peptides, as these peptides were designed to be specific for human MHC-II.	Very high. Reduced pulmonary fungal burden by 3–4 log CFU; high survival following severe aerosol challenge.	Genus-level cross-protection. Induced robust immunity against both *C. posadasii* and *C. immitis*.
Thermally Dimorphic group: Blastomyces /Histoplasma /Coccidioides [12]	Endoglucanase-2 (Eng2) homologues	TVWFFPRGNIPDD (*C. posadasii*-Eng2), NFFNGPDPSNGYV (*H. capsulatum*-Eng2)	- The restriction is specific to MHC class II, which primarily presents antigens to activate CD4 T cells. - In experimental animals: Inbred C57BL/6 mice (through the MHC class II molecule I-Aᵇ). Humanized HLA-DR4 transgenic mice (DRB1*0401), which express human MHC class II molecules in place of mouse MHC molecules. In humans: Antigen presentation occurs through various HLA class II molecules (HLA-DRB1 haplotypes).	CD4⁺ TH1/TH17	**- MHC Class II Tetramer Staining and Pulldown:** Generate and use peptide–MHC class II tetramers to track, quantify, and confirm the specificity of Eng2-specific CD4 T cells. **- Intracellular Cytokine Staining (ICS) and ELISA:** Measure the production of IFN-γ, IL-17A, and other cytokines following repeated stimulation with antigens or peptides. **- Sanofi’s MIMIC System:** Use monocyte-derived dendritic cells to present antigens to naïve human CD4+ T cells to confirm T-cell priming and their ability to respond. Vaccination and Intratracheal Challenge: Vaccinate C57BL/6 and HLA-DR4 mice subcutaneously with Eng2 protein or peptides formulated in glucan–chitin particles (GCPs) or mixed with an adjuvant, followed by an intratracheal fungal challenge.	- **Cp-Eng2 against *C. posadasii*:** Reduced fungal burdens in the lungs and spleen by up to 3–4 logs in CFU, prevented weight loss, and significantly improved survival rates. - **Hc-Eng2 against *H. capsulatum*:** Reduced CFU in the lungs and spleen and significantly improved survival rates. - **Bl-Eng2 against *B. dermatitidis*:** Reduced CFU in the lungs and protected against severe infection. - **Single-Peptide Vaccines:** Vaccination with individual peptides, such as Cp-Eng2 Peptide 3 or Bl-Eng2 Peptide 3, achieved reductions in lung CFU comparable to those obtained with the full-length protein.	**- In Vivo Cross-Protection:** No cross-protection was observed. Vaccination with Eng2 from one fungal species did not protect against infection by another fungal species (e.g., Bl-Eng2 did not protect against *C. posadasii* or *H. capsulatum*), because the immunodominant T-cell epitopes in each homolog differ in sequence and location. **- Specificity and Cross-Reactivity in Humans:** In patients who have recovered from infection, cross-reactive CD4+ T-cell recall responses to Eng2 homologs from other fungal species have been observed to some extent. These responses are attributable to shared subdominant epitopes, even though these are not the major protective epitopes identified in animal models. This cross-reactivity suggests potential for developing a pan-fungal diagnostic tool.

### 6.4. Development of a rCpa1 Vaccine Candidate Against Coccidioidomycosis

Perhaps the most sophisticated application of MHC restriction principles in antifungal vaccinology is exemplified by the recombinant chimeric polypeptide antigen 1 (rCpa1) vaccine developed against *C. posadasii* and *C. immitis* [70]. Recognizing that a single antigen is insufficient to overcome pathogen diversity, investigators engineered a multivalent recombinant construct incorporating the three most immunogenic fungal antigens previously identified (Ag2/Pra, Cs-Ag, and Pmp1), together with five promiscuous CD4 T-cell epitopes predicted to bind multiple human HLA class II molecules.

The five promiscuous epitopes included: Pep1-P1 (MRNSILLAATVLLGCTSAKVH), Pep1-P2 (HVRALGQKYFGSLPSSQQQTV), Amn1-P9 (PAKVDVLLAQSLKLADVLKF), Amn1-P10: (NGLATTGTLVLEWTRLSDIT) and Plb-P6 (TPLVVYIPNYPYTTWSNIST) [71].

Bioinformatic prediction algorithms, such as ProPred, predicted that these specific sequences, particularly Plb-P6 derived from phospholipase B, exhibit exceptionally high-affinity binding to multiple human HLA-DR alleles [84]. To prevent the inadvertent generation of junctional neo-epitopes during intracellular processing, vaccine engineers incorporated glycine-proline (GPGPG) spacers at the C-terminus of each peptide linked within the rCpa1 construct [71,85]. To evaluate its efficacy, rCpa1 was encapsulated in GCPs, which functioned as an innate adjuvant to target PRRs on APCs, and was administered to immunocompetent C57BL/6 mice and transgenic mice expressing human HLA-DR4. Immune responses were monitored by flow cytometry using fluorescent markers, including FITC-CD45, APC-Fire750-CD4, and BV421-CD44, in combination with intracellular cytokine staining. These analyses provided important insights, demonstrating that vaccination strongly induced HLA-DR4-restricted memory TH1 (IFN-γ) and TH17 (IL-17) cell populations. This response significantly reduced fungal burdens in the lungs and protected the animals against severe infection caused by diverse clinical strains of both *C. posadasii* and *C. immitis*, despite the presence of seven amino acid substitutions in the rCpa1 construct between the two species [70,71]. Overall, these findings demonstrate that rational vaccine design integrating promiscuous HLA class II epitopes with targeted innate immune activation can overcome genetic diversity and induce broad, durable antifungal immunity.

With regard to their synergistic adjuvant properties, β-Glucan and chitin are major structural polysaccharides of the fungal cell wall that function as pathogen-associated molecular patterns (PAMPs). These molecules are recognized by PRRs expressed on innate immune cells, initiating or modulating antifungal inflammatory responses [86]. Among these receptors, Dectin-1 serves as the principal C-type lectin receptor that specifically recognizes β-1,3-glucans on fungal surfaces. Toll-like receptors (TLR-2 and TLR-4) frequently cooperate with Dectin-1 to amplify downstream signaling, whereas mannose receptors and LYSMD3, expressed on macrophages and epithelial cells, contribute to the recognition of chitin and other fungal cell wall components. Engagement of these PRRs activates intracellular signaling pathways that promote the production of pro-inflammatory cytokines, including TNF-α, IL-17, IL-18, and IL-23, thereby enhancing antifungal immunity. Importantly, simultaneous recognition of β-glucan and chitin can synergistically potentiate inflammatory responses. However, the immunological effects of these polysaccharides are context-dependent; variations in their molecular size, structural characteristics, and purity may shift the immune response from robust pro-inflammatory activation toward anti-inflammatory or regulatory-manipulation pathways, revealing their dual immunomodulatory properties [87,88,89].

## 7. Broader Implications and Second Order Insights

Although evidence addressing these issues remains limited, the synthesis of the currently available knowledge on MHC binding/restriction in antifungal immunity provides important primary and secondary insights that establish a foundation for the development of future immunotherapeutic and vaccine-based strategies.

One of the most important lessons derived from studies of the Eng2 and Crf1 antigens is that amino acid sequence homology alone does not guarantee cross-protective immunity. Although the genus Aspergillus and genus Candida are evolutionarily distant and morphologically distinct fungi, the specific proteolytic processing of the Crf1 protein generates a peptide (p41) with remarkably high structural similarity that can be presented by human HLA class II molecules in a conformation permissive for TCR cross-reactivity [79]. In contrast, among dimorphic fungal pathogens, despite the overall structural conservation of Eng2 homologues, the immunodominant epitopes are positioned in entirely different regions of the protein depending on the fungal species. This observation suggests that even subtle amino acid substitutions outside the epitope core may profoundly alter cathepsin-mediated proteolytic cleavage within APCs, thereby reshaping the repertoire of peptides available for MHC presentation and ultimately abolishing cross-reactive T-cell recognition. Consequently, the development of pan-fungal vaccines cannot rely solely on bioinformatic predictions of sequence conservation but must incorporate empirical epitope mapping together with cross-species MHC-binding validation.

Another issue that warrants careful reconsideration is the long-standing paradigm of antifungal immunity, which has traditionally emphasized CD4 TH1 and TH17 responses. Emerging evidence demonstrating that exogenous fungal antigens can activate CD8 T cells through MHC class I restriction in the absence of CD4 T-cell help fundamentally challenges this conventional view [13]. Because fungal pathogens generally do not invade the cytosol of APCs, this process depends almost entirely on the efficiency of DC-mediated cross-presentation [90]. The discovery that Asp f16-specific CTLs, restricted by HLA-B*3501, can directly damage fungal hyphae and inhibit conidial growth suggests that future antifungal immunotherapies and vaccines should intentionally incorporate CD8 T-cell epitopes to elicit coordinated CD4 and CD8 cellular immunity. Exploiting this cross-presentation pathway may be particularly valuable in clinical settings characterized by profound CD4 T-cell depletion, such as advanced HIV/AIDS or post-transplant immunosuppression [91].

The success of the recombinant Cpa1 (rCpa1) vaccine further highlights the critical intersection between innate immune recognition and MHC-restricted acquired immunity. Beyond the role of natural IgM in facilitating fungal antigen trafficking to draining lymph nodes and establishing a favorable microenvironment for T-cell priming, encapsulation of rCpa1 epitopes within glucan-chitin particles (GCPs) substantially enhances TH17 responses compared with administration of soluble antigen alone [71,92,93]. β-Glucan and chitin directly engage innate PRRs, particularly Dectin-1, on APCs, thereby promoting the production of cytokines such as IL-6, TGF-β, and IL-23 that polarize MHC-restricted CD4 T cells toward the TH17 lineage, a subset indispensable for antifungal defense [94,95]. These findings suggest that optimal antifungal T-cell immunity depends not only on accurately matching peptide epitopes with the host HLA repertoire but also on delivering those antigens within an appropriate innate inflammatory context that supports effective antigen presentation and T-cell differentiation.

Finally, persistent presentation of fungal antigens by MHC molecules during chronic or disseminated fungal infections, such as disseminated coccidioidomycosis, frequently drives antigen-specific T cells into a state of functional exhaustion. Exhausted T cells progressively upregulate inhibitory receptors, including PD-1, while APCs may concomitantly increase expression of their corresponding ligands. In vitro studies have demonstrated that supplementation with PD-1 blocking antibodies restores the proliferative capacity and cytokine-producing function of fungus-specific T cells [66]. These observations provide a compelling rationale for repurposing immune checkpoint inhibitors, which have emerged as key components of modern cancer immunotherapy, as adjunctive treatments to reinvigorate MHC-restricted antifungal T-cell responses in patients with refractory or drug-resistant invasive fungal infections.

## 8. Summary and Future Perspectives

The principle of MHC-restricted antigen recognition represents a fundamental paradigm governing acquired immunity to fungal pathogens. During fungal infection, protein antigens are processed into peptide epitopes and presented by MHC molecules to antigen-specific T cells, thereby orchestrating coordinated cellular and humoral immune responses. While polysaccharide antigens such as cryptococcal glucuronoxylomannan (GXM) are recognized directly by antibodies rather than through MHC-dependent pathways, their biological significance has driven major advances in translational immunology. For example, the successful development of an in-house immunochromatographic assay utilizing monoclonal antibody 18B7 for GXM detection demonstrates how detailed knowledge of fungal antigenicity can be translated into highly accurate diagnostic platforms [96]. Although antibody-based immunodiagnostics operate independently of MHC-mediated antigen presentation, they exemplify the broader principle that mechanistic understanding of host–pathogen interactions provides the foundation for innovation in clinical medicine.

Over the past several decades, the study of MHC function in antifungal immunity has progressed from a fundamental immunological concept to a precisely defined molecular discipline. The integration of overlapping peptide libraries, ELISPOT assays, intracellular cytokine profiling, antibody-mediated blocking experiments, proliferation assays, and peptide–MHC multimer technologies has enabled the systematic identification and functional characterization of immunodominant fungal T-cell epitopes. These complementary approaches have transformed our understanding of antigen processing and presentation, allowing fungal-specific T-cell responses to be analyzed with unprecedented molecular and cellular resolution.

This expanding body of evidence has identified numerous immunodominant antigens—including Crf1, Asp f16, Eng2, and the recombinant chimeric vaccine candidate rCpa1—that elicit potent TH1-, TH17-, and CD8 CTL responses. Several of these antigens have demonstrated varying degrees of cross-protective immunity against phylogenetically distinct fungal pathogens, emphasizing the feasibility of developing broadly protective antifungal vaccines. Importantly, these immune responses are restricted by well-characterized human HLA alleles, and their validation in human HLA-transgenic mouse models has provided a critical bridge between mechanistic immunology and clinical translation [97]. Looking forward, continued advances in immunopeptidomics, single-cell multiomics, spatial transcriptomics, artificial intelligence-assisted epitope prediction, and structural biology or chemistry are expected to further refine our understanding of fungal antigen processing, peptide–MHC binding, and T-cell receptor recognition at atomic and systems levels [92,98,99,100]. These technologies will facilitate the discovery of conserved immunodominant epitopes across diverse fungal species while accounting for the remarkable genetic diversity of human HLA molecules. Such knowledge will accelerate the development of personalized and broadly protective antifungal vaccines, precision immunodiagnostic platforms, and next-generation cellular immunotherapies. Ultimately, deciphering the molecular rules that govern the processing, presentation, and recognition of fungal antigens represents far more than an academic pursuit. It provides the conceptual framework for rational vaccine design, T-cell receptor engineering, adoptive T-cell therapy, and other precision immunotherapeutic strategies. As invasive fungal diseases continue to emerge as a major global health challenge—particularly among immunocompromised individuals—translating the principles of MHC restriction into clinically actionable interventions will be essential for improving diagnosis, prevention, and treatment, thereby shaping the future of antifungal medicine.

T-cell recognition and MHC restriction represent fundamental mechanisms in antifungal prophylaxis and vaccine design. The delay in vaccine development stems from complex and interconnected barriers ranging from the molecular level and pathogen immune evasion to host genetic diversity and limitations in animal model simulation. Together, these factors undermine the successful translation of research findings into clinical applications. A primary barrier is the blind spot in antigen prediction systems and fungal immune evasion. Traditional bioinformatics approaches to epitope prediction often fail because their algorithms do not account for post-translational modifications, which are crucial for fungal survival and pathogenesis. Pathogenic fungi rely heavily on glycosylation and phosphorylation to modify protein structures. For instance, fungal glycans are typically rich in densely packed mannans that mask antigens from detection by the human immune system. Disruption of this process, such as through inhibition of the alpha 1,6 mannosyltransferase (Och1) enzyme, significantly weakens the fungal cell wall and reduces virulence. Furthermore, phosphorylation mediated by kinases such as Tpk2 and Pka1 acts as a signaling switch to rapidly regulate fungal morphology and capsule formation [101]. These factors can cause computationally optimized antigen targets to fail to elicit authentic immune responses in vivo within the human population. To overcome this limitation, researchers must turn to immunopeptidomics technology, employing advanced analytical tools such as LC-MS/MS to extract and identify peptides that evade shielding and are genuinely presented on HLA molecules under natural infection conditions [102].

Beyond fungal antigen shielding, research must also address human genetic diversity and immunological bottlenecks. The human HLA gene system exhibits immense polymorphism, with more than 35,000 distinct alleles identified [103]. Developing a vaccine capable of providing broad population coverage requires the identification of epitopes that can bind to diverse HLA molecules. Moreover, a critical factor undermining vaccine success is that individuals at high risk of fungal infections may harbor specific genetic defects. A prominent example is a mutation in the CLEC7A gene that results in the Dectin 1 Y238X variant, which causes the receptor to lose an essential domain required for recognizing beta-glucan structures in the fungal cell wall. This leads to diminished TNF-α production and a substantially increased risk of invasive Candida and Aspergillus infections [104]. Similarly, CARD9 deficiency impairs the ability of macrophages to respond to and contain fungal pathogens, rendering affected individuals susceptible to subcutaneous fungal infections such as those caused by *Phialophora verrucosa*. These genetic impairments can compromise the development of TH17 responses, which constitute a central component of antifungal defense [105].

The limitations of animal models constitute another factor that can distort vaccine efficacy assessments. Historically, preclinical data have often been derived from vaccine testing in mice that have been chemically immunosuppressed to facilitate infection. This approach can inadvertently impair the very immune cells upon which vaccines depend to generate immunological memory against pathogens. Consequently, research is increasingly moving toward humanized mouse models genetically engineered to express human HLA molecules, such as strains carrying the HLA-DR4 gene. These models can provide a more human-relevant platform for investigating antigen-specific immune responses and antibody production. This approach can be complemented by organoid technology and organ-on-a-chip-based microfluidic systems, which enable researchers to simulate and observe Candida invasion across endothelial cell barriers at high cellular resolution. These platforms help address the limitations of conventional animal models and provide more physiologically relevant insights into host–pathogen interactions.

Although several vaccine candidates have already advanced into clinical trials, including the NDV 3A vaccine targeting the Candida agglutinin-like sequence 3 (Als3) protein and the PEV7 vaccine targeting the secreted aspartyl proteinase 2 (Sap2) protein [82], the research community continues to accelerate the development of advanced technologies such as mRNA lipid nanoparticle (mRNA-LNP) vaccines. These platforms offer the potential to circumvent some manufacturing complexities associated with large glycoproteins. Their RNA components can also be modified using nucleoside analogues such as N1-methylpseudouridine to modulate innate immune recognition and improve tolerability. Such vaccine platforms may be combined with novel adjuvants, including Cationic Adjuvant Formulation 01 (CAF01), which is designed to engage the C-type lectin receptor Mincle and promote robust TH1 and TH17 memory responses [106,107]. Furthermore, the concept of trained immunity, which involves using compounds such as beta-glucan to induce epigenetic reprogramming of innate immune cells, is attracting increasing interest as a potential strategy for providing temporary prophylactic protection to high-risk patients before they enter immunocompromised states [108].

Ultimately, research on antigen cross-presentation and alternative T-cell pathways is closely aligned with UN-SDG 3 (Good Health and Well-Being), as it directly addresses the prevention and treatment of life-threatening infections in highly vulnerable populations [109]. By elucidating cross-presentation pathways that can activate protective CD8 T-cell responses independently of conventional CD4 T-cell help, this body of research may contribute to the development of innovative therapeutic vaccines and immunotherapies for immunocompromised patients. Furthermore, investigations into fungal antigen cross-presentation provide fundamental biomedical knowledge that supports the research and development of next-generation vaccines and treatments for communicable diseases, particularly in patients who respond poorly to conventional therapeutic approaches. Finally, given the extensive use of one-letter amino acid codes throughout the text, a corresponding list of amino acid names and their respective codes is provided in Appendix A [110].

## Data Availability

No new data were created or analyzed in this study. Data sharing is not applicable to this article.

## Abbreviations

The following abbreviations are used in this manuscript:

aAPCs	Amateur Antigen-Presenting Cells
APCs	Antigen-Presenting Cells
CARD9	Caspase recruitment domain containing protein 9
cDC1s	Conventional Dendritic Cells type 1
cDC2s	Conventional Dendritic Cells type 2
CFSE	Carboxyfluorescein Succinimidyl Ester
CLIP	Class II-Associated Invariant Chain Peptide
cTECs	Cortical Thymic Epithelial Cells
CTLs	Cytotoxic T Lymphocytes
DCs	Dendritic cells
ELISpot	Enzyme-Linked ImmunoSpot
GCPs	Glucan–Chitin Particles
GM-CSF	Granulocyte–Macrophage Colony-Stimulating Factor
GXM	Glucuronoxylomannan
HLA	Human Leukocyte Antigen
iNOS	Inducible Nitric Oxide Synthase
LC-MS/MS	Liquid chromatography–tandem mass spectrometry
MAIT	Mucosal-Associated Invariant T Cells
MHC	Major Histocompatibility Complex
MIMIC	Modular Immune In vitro Construct
MR1	Major Histocompatibility Complex Class I-Related Protein
mTECs	Medullary Thymic Epithelial Cells
PAMPs	Pathogen-Associated Molecular Patterns
pAPCs	Professional Antigen-Presenting Cells
PBMCs	Peripheral Blood Mononuclear Cells
PD-1	Programmed Death Protein 1
pMHC	Peptide–MHC
PRRs	Pattern Recognition Receptors
rMp1p	Recombinant Mannoprotein 1 of Talaromyces marneffei
TAP	Transporter Associated with Antigen Processing
TCR	T cell Receptor
TFH	T follicular helper cells
TLRs	Toll-Like Receptors
WHO-FPPL	World Health Organization Fungal Priority Pathogens List

## Appendix A

The 20 standard amino acids with their 1-letter code, 3-letter abbreviation, and the full name [110].

**1-Letter**	**3-Letter**	**Full Name**
**A**	Ala	Alanine
**R**	Arg	Arginine
**N**	Asn	Asparagine
**D**	Asp	Aspartic acid
**C**	Cys	Cysteine
**E**	Glu	Glutamic acid
**Q**	Gln	Glutamine
**G**	Gly	Glycine
**H**	His	Histidine
**I**	Ile	Isoleucine
**L**	Leu	Leucine
**K**	Lys	Lysine
**M**	Met	Methionine
**F**	Phe	Phenylalanine
**P**	Pro	Proline
**S**	Ser	Serine
**T**	Thr	Threonine
**W**	Trp	Tryptophan
**Y**	Tyr	Tyrosine
**V**	Val	Valine
